# Ethnicity, Child Sex, and the Likelihood of Marriage in Pregnancy: A Novel Analysis of Gender Inequity

**DOI:** 10.3389/ijph.2022.1604869

**Published:** 2022-09-06

**Authors:** Nathalie Auger, Clara Bolster-Foucault, Marianne Bilodeau-Bertrand, Sahar Khademi, Améyo Djeha

**Affiliations:** ^1^ University of Montreal Hospital Centre (CRCHUM), Montreal, QC, Canada; ^2^ Department of Epidemiology, Biostatistics and Occupational Health, School of Population and Global Health, Faculty of Medicine and Health Sciences, McGill University, Montreal, QC, Canada; ^3^ Institut national de santé publique du Québec, Québec, QC, Canada; ^4^ Department of Social and Preventive Medicine, School of Public Health, University of Montreal, Montreal, QC, Canada

**Keywords:** pregnancy, cultural minorities, gender inequity, marriage, son preference

## Abstract

**Objective:** We assessed the association between fetal sex and the likelihood of marriage during pregnancy.

**Methods:** We analyzed a cohort of 1,334,911 women who were unmarried at conception and had a live birth between 1990 and 2018 in Quebec, Canada. The exposure was fetal sex, determined by ultrasound. The outcome was marriage during pregnancy. We estimated hazard ratios and 95% confidence intervals (CI) for the association of child sex with the likelihood of marriage during pregnancy according to region of origin.

**Results:** Among women who were unmarried at conception, 13.1% of foreign-born women got married during pregnancy compared with 2.6% of Canadian-born women. Women from the Middle East and North Africa who were pregnant with boys were 1.13 times more likely to marry during pregnancy compared with women who were pregnant with girls (95% CI 1.02–1.26). There was no association among Canadian-born women.

**Conclusion:** Women from some cultural minorities who are pregnant with boys may be more likely to marry during pregnancy in Western settings. Gender inequality may manifest as a preference for sons that influences the likelihood of marriage.

## Introduction

Many unmarried women marry while pregnant, but the sex of the child is an underrecognized factor that may influence the likelihood of marriage. Son preference is common in many cultures and is known to affect prenatal decisions during pregnancy [1–5]. In some cultures, women who are pregnant with girls opt for sex-selective abortion [1–5], or in extreme cases, infanticide [3]. Some cultural groups may have more pronounced son preference due to family pressure, cultural and religious traditions, family name propagation, need to protect the marriage, and socioeconomic responsibilities [1–4, 6]. Because the sex of a child can have a strong influence on the decision to pursue a pregnancy, it is possible that unmarried pregnant women who are carrying boys may be more likely to marry because these pregnancies may be perceived as more valuable.

Marital status and family composition are well-established determinants of maternal and infant health [7, 8]. Unmarried pregnant women have an elevated risk of low birth weight, preterm birth, and small-for-gestational-age at birth after controlling for ethnicity [7], as well as a higher prevalence of postpartum depression [8]. Children of unmarried or single parents are at risk of obesity, asthma, injury, and hospitalization, as well as behavioural problems and suboptimal cognitive development [9]. Marriage during pregnancy has the potential to improve maternal and child outcomes, but the factors that influence the likelihood of marriage are not understood. Gender inequity is a widely recognized determinant of health [10]. Investigating the association between ethnicity, child sex, and marriage during pregnancy is important because identifying gender inequity in Western settings may be challenging using usual indicators. Marriage during pregnancy is a potential indicator of son preference in Western countries, where gender inequity related to pregnancy is not well understood. The objective of this study was to assess the relationship between child sex and the likelihood of marriage during pregnancy in a multicultural Western setting where son preference has been documented through sex-selective abortion [11].

## Methods

### Study Design

We used a retrospective cohort design to study women who were unmarried at the start of pregnancy and had a live birth, girl or boy, any time between 1990 and 2018 in Quebec, Canada. We began the study in 1990 because routine ultrasounds were implemented in Quebec in the early 1990s, allowing parents to determine the sex of their child in the first or second trimester [12].

We acquired information from birth registration certificates. Birth certificates collect data on marital status, date of marriage, country of birth, mother tongue, child sex, and gestational age. We used the mother tongue and country of birth as markers of cultural status. We excluded women who were already married at the time of conception, had infants of undetermined sex, or had missing gestational age, mother tongue, or country of birth.

### Exposure

The exposure of interest was child sex, as documented on birth certificates. During the study, parents were covered by publicly funded prenatal healthcare that included ultrasound in the first and second trimesters of pregnancy. Ultrasound can reliably detect fetal sex towards the end of the first trimester, at around 12 weeks of gestation [13]. Visualization of the sagittal sign at 14 weeks can determine fetal sex with nearly 100% accuracy [13]. Non-invasive prenatal testing with cell-free DNA can determine sex as early as 7 weeks [14], although these tests are used less frequently.

In Quebec, women routinely have dating ultrasounds at 11–13 weeks and birth defect screening ultrasounds at 18–22 weeks [15, 16]. The majority of women are informed of the sex of their child during ultrasound. The proportion of women who refuse to know the sex, or ultrasound technicians who fail to visualize the sagittal sign or external genitalia, is expected to be low.

### Outcome

The outcome was marriage during pregnancy. We had the exact date of marriage, as well as the date and gestational age at birth. We estimated the conception date by subtracting the gestational age from the date of birth and determined the number of days between conception and the date of marriage. Follow-up began at conception and ended at the time of marriage or birth. We censored all women who did not marry before the birth of their child.

### Covariates

We considered cultural factors that could modify the relationship between child sex and the likelihood of marriage. We used country of birth and mother tongue as indicators of culture in this analysis. Previous studies indicate that both country of birth and language are associated with perinatal outcomes in Quebec [17, 18].

We classified country of birth following World Bank regions [11], including Middle East and North Africa, East Europe, South Asia, East Asia and the Pacific, West Europe and the US, Sub-Saharan Africa, Latin America, the Caribbean, and Canada. As Quebec is a Francophone province with minority Anglophone and foreign-language populations, we classified women as having French, English, or a foreign mother tongue. We grouped all foreign languages and also analyzed linguistic groups separately. We used French and English as the comparison group because these two linguistic populations share cultural characteristics. Son preference is not prominent among Francophones or Anglophones in Quebec, and a large proportion of both live as common-law couples without formally marrying [19].

We accounted for confounders such as age at delivery (<25, 25–34, ≥35 years), primiparity, and education (no high school diploma, high school diploma or postsecondary training, university degree, unknown). Age at delivery, primiparity, and education were collected by self-report in birth certificates. Because such factors can be linked with cultural status but not the sex of a child at conception, we included these covariates as confounders of the association between cultural status and marriage, but not child sex and marriage. We did not have data on the use of sex-selective abortion. However, we stratified analyses by cultural groups where sex-selective abortion has been documented [3]. Previous analyses of Quebec data indicate that mothers of Indo-Pakistani descent had a higher sex ratio than expected in the past, suggesting the possibility of sex selective abortion [11].

### Data Analysis

We computed marriage rates stratified by region of origin and mother tongue and plotted the cumulative percentage by gestational age. In primary analyses, we used Cox regression to obtain hazard ratios (HR) with 95% confidence intervals (CI) for the association of cultural group with the probability of marriage during pregnancy. We adjusted these models for age at delivery, parity, and education. We subsequently used Cox regression to estimate the association between child sex and the probability of marriage in each region of origin and language group separately.

We examined the association of child sex with marriage anytime during pregnancy, beginning follow-up at conception and ending at marriage (event) or delivery (censored). Because ultrasound is less reliable at determining sex before 12 weeks, we expected that child sex would be more weakly associated with marriage before this time point. We also expected stronger associations after the second trimester ultrasound at 18 or 22 weeks when a larger proportion of women would know the child’s sex. We therefore performed analyses in which we stopped follow-up at 12 weeks, and analyses in which follow-up began at 12, 18, and 22 weeks of pregnancy.

In sensitivity analyses, we assessed whether associations differed when we restricted the analysis to the first pregnancy only. We also stratified the analysis by period (1990–1999, 2000–2009, 2010–2018), as new technologies may have facilitated earlier determination of sex over time and secular trends in son preference may have changed during the 30-year study period. We analyzed the data using SAS v9.4 (SAS Institute Inc., Cary, NC). As the data were anonymous, consent to participate was not required. The institutional review board of the University of Montreal Hospital Centre waived ethics review.

## Results

There were 1,334,911 women who were unmarried at the start of pregnancy, including 45,842 women with a foreign mother tongue and 80,295 women whose region of origin was outside of Canada (Table 1). Overall, only 2.8% of French and English speakers got married during pregnancy, compared with 15.9% of women with a foreign mother tongue. Similarly, only 2.6% of Canadian-born women got married during pregnancy. In contrast, between 8.7% and 30.3% of foreign-born women got married during pregnancy. Marriage during pregnancy was most frequent among women from the Middle East and North Africa (30.3%). Women with a foreign language were more likely to be ≥ 35 years and have no high school diploma compared with French and English-speaking women (Table 2).

**TABLE 1:** Association of mother tongue and region of birth with marriage during pregnancy, Canada, 1990–2018.

	No. women who married during pregnancy (%)	Total no. women	Hazard ratio (95% confidence interval)	
			Unadjusted	Adjusted^a^
All women	42,899 (3.2)	1,334,911	—	—
Mother tongue				
Foreign language	7,304 (15.9)	45,842	6.16 (6.01–6.32)	6.31 (6.16–6.48)
French or English	35,595 (2.8)	1,289,069	Reference	Reference
Region of origin				
Middle East and North Africa	1,351 (30.3)	4,457	13.73 (13.00–14.50)	12.62 (11.94–13.34)
Eastern Europe	1,000 (21.0)	4,755	8.80 (8.26–9.37)	7.52 (7.06–8.01)
South Asia	291 (23.8)	1,225	10.35 (9.22–11.62)	11.47 (10.22–12.87)
East Asia and Pacific	1,739 (18.6)	9,355	7.80 (7.44–8.19)	7.66 (7.29–8.04)
West Europe and United States	1,701 (9.4)	18,085	3.75 (3.57–3.93)	3.30 (3.14–3.46)
Sub-Saharan Africa	1,036 (12.1)	8,560	4.91 (4.62–5.23)	4.86 (4.57–5.17)
Latin America	1,556 (12.1)	12,916	4.87 (4.63–5.12)	5.08 (4.82–5.34)
Caribbean	1,815 (8.7)	20,942	3.47 (3.31–3.63)	3.94 (3.76–4.14)
Canada	32,410 (2.6)	1,254,616	Reference	Reference

a Adjusted for age at delivery, parity, and education.

**TABLE 2:** Characteristics of women at delivery by mother tongue and region of origin, Canada, 1990–2018.

	No. women (%) N = 1,334,911					
	Age at delivery			Education		
	<25 years	25–34 years	≥35 years	No high school diploma	High school diploma or postsecondary training	University training
Mother tongue						
Foreign language	11,646 (25.4)	24,167 (52.7)	10,029 (21.9)	8,040 (17.5)	23,894 (52.1)	10,774 (23.5)
French or English	347,147 (26.9)	800,775 (62.1)	141,147 (10.9)	152,712 (11.8)	740,636 (57.5)	337,700 (26.2)
Region of origin						
Middle East and North Africa	616 (13.8)	2,382 (53.4)	1,459 (32.7)	319 (7.2)	1,733 (38.9)	1,906 (42.8)
East Europe	899 (18.9)	2,648 (55.7)	1,208 (25.4)	245 (5.2)	1,922 (40.4)	2,292 (48.2)
South Asia	325 (26.5)	713 (58.2)	187 (15.3)	277 (22.6)	701 (57.2)	147 (12.0)
East Asia and Pacific	1,532 (16.4)	5,453 (58.3)	2,370 (25.3)	1,437 (15.4)	4,635 (49.5)	2,597 (27.8)
West Europe and United States	2,489 (13.8)	11,394 (63.0)	4,202 (23.2)	1,041 (5.8)	7,102 (39.3)	9,173 (50.7)
Sub-Saharan Africa	1,724 (20.1)	5,102 (59.6)	1,734 (20.3)	875 (10.2)	4,191 (49.0)	2,803 (32.7)
Latin America	3,904 (30.2)	6,511 (50.4)	2,501 (19.4)	2,373 (18.4)	6,875 (53.2)	2,988 (23.1)
Caribbean	5,667 (27.1)	10,743 (51.3)	4,532 (21.6)	4,454 (21.3)	12,469 (59.5)	2,587 (12.4)
Canada	341,637 (27.2)	779,996 (62.2)	132,983 (10.6)	149,731 (11.9)	724,902 (57.8)	323,981 (25.8)

In adjusted regression models, mother tongue and region of origin were both associated with the probability of marriage regardless of child sex (Table 1). Compared with French and English-speaking women, women with foreign mother tongues were 6.31 times more likely to marry during pregnancy (95% CI 6.16–6.48). Women from all foreign regions of origin were more likely to marry, although associations were stronger for some regions. Women who originated from the Middle East and North Africa were 12.62 times more likely to marry (95% CI 11.94–13.34) and women from South Asia were 11.47 times more likely to marry during pregnancy (95% CI 10.22–12.87), compared with Canadian-born women.

In most cultural groups, there was no clear association between child sex and the overall probability of marriage during pregnancy (Table 3). However, women from the Middle East and North Africa who were pregnant with boys were 1.13 times more likely to marry compared with women from the same region who were pregnant with girls (95% CI 1.02–1.26). Women from Eastern Europe also had a greater tendency to marry when they were pregnant with boys (HR 1.11, 95% CI 0.98–1.26).

**TABLE 3:** Association between child sex and marriage during pregnancy according to mother tongue and region of origin, Canada, 1990–2018.

	Women pregnant with boys		Women pregnant with girls		Hazard ratio boys vs girls (95% confidence interval)
	No. who married during pregnancy (%)	Total no. women	No. who married during pregnancy (%)	Total no. women	
All women	22,102 (3.2)	685,067	20,797 (3.2)	649,844	1.01 (0.99–1.03)
Mother tongue					
Foreign language	3,764 (16.2)	23,302	3,540 (15.7)	22,540	1.03 (0.98–1.08)
French or English	18,338 (2.8)	661,765	17,257 (2.8)	627,304	1.01 (0.99–1.03)
Region of origin					
Middle East and North Africa	735 (31.8)	2,311	616 (28.7)	2,146	1.13 (1.02–1.26)
Eastern Europe	530 (22.0)	2,407	470 (20.0)	2,348	1.11 (0.98–1.26)
South Asia	154 (23.9)	645	137 (23.6)	580	1.01 (0.80–1.27)
East Asia and Pacific	897 (18.7)	4,798	842 (18.5)	4,557	1.02 (0.93–1.12)
West Europe and United States	878 (9.5)	9,205	823 (9.3)	8,880	1.03 (0.94–1.14)
Sub-Saharan Africa	523 (12.1)	4,332	513 (12.1)	4,228	0.99 (0.88–1.12)
Latin America	803 (12.0)	6,680	753 (12.1)	6,236	0.99 (0.90–1.10)
Caribbean	913 (8.7)	10,511	902 (8.7)	10,431	1.01 (0.92–1.10)
Canada	16,669 (2.6)	644,178	15,741 (2.6)	610,438	1.00 (0.98–1.03)

**FIGURE 1:**
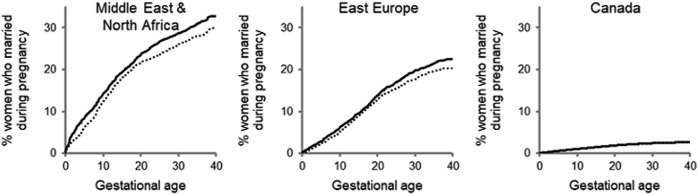
Cumulative frequency of marriage during pregnancy according to region of origin, Canada, 1990–2018.^a^Solid line denotes women pregnant with boys; dotted line denotes women pregnant with girls. Solid and dotted lines overlap for women originating from Canada. Gray *p* value: Middle East and North Africa 0.02; East Europe 0.09; Canada 0.7.

These patterns became more pronounced when we considered the gestational age and timing of routine ultrasounds that informed parents of the child’s sex (Figure 1). After 18 weeks of gestation, women from the Middle East and North Africa who were pregnant with boys had a higher cumulative frequency of marriage than women who were pregnant with girls. At 22 weeks, the cumulative frequency of marriage was 24.6% (95% CI 22.8–26.4) for women pregnant with boys compared with 20.4% (95% CI 20.3–23.8) for women pregnant with girls. Women from Eastern Europe exhibited similar trends. No such difference was apparent among Canadian-born women.

The association between child sex and the probability of marriage strengthened somewhat over the course of the second trimester, when a greater proportion of women likely knew the child’s sex (Table 4). Women from the Middle East and North Africa who were pregnant with boys had 1.11 times the likelihood of marriage after 12 weeks (95% CI 0.95–1.29) and 1.26 times the likelihood of marriage after 18 weeks of pregnancy (95% CI 1.04–1.53), compared with women from the same region who were pregnant with girls. Child sex was not associated with marriage among women from South Asia, East Asia, West Europe and the United States, or Canada at any gestational age.

**TABLE 4:** Association of child sex with gestational age at marriage according to region of origin, Canada, 1990–2018.

	No. women who married during pregnancy (%)		Hazard ratio boys vs girls (95% confidence interval)
	Pregnant with boys N = 22,102	Pregnant with girls N = 20,797	
Middle East and North Africa			
<12 weeks	327 (14.2)	262 (12.2)	1.16 (1.00–1.35)
≥12 weeks	408 (17.7)	354 (16.5)	1.11 (0.95–1.29)
≥18 weeks	267 (11.6)	214 (10.0)	1.26 (1.04–1.53)
≥22 weeks	193 (8.4)	149 (6.9)	1.18 (0.94–1.47)
Eastern Europe			
<12 weeks	149 (6.2)	121 (5.2)	1.12 (0.91–1.39)
≥12 weeks	381 (15.8)	349 (14.9)	1.11 (0.95–1.29)
≥18 weeks	282 (11.7)	241 (10.3)	1.15 (0.96–1.38)
≥22 weeks	197 (8.2)	169 (7.2)	1.16 (0.92–1.45)
South Asia			
<12 weeks	61 (9.5)	59 (10.2)	0.94 (0.67–1.31)
≥12 weeks	93 (14.4)	78 (13.5)	1.08 (0.79–1.49)
≥18 weeks	56 (8.7)	54 (9.3)	0.94 (0.63–1.41)
≥22 weeks	39 (6.1)	41 (7.1)	0.99 (0.61–1.60)
East Asia and Pacific			
<12 weeks	337 (7.0)	279 (6.1)	1.12 (0.97–1.30)
≥12 weeks	560 (11.7)	563 (12.4)	0.95 (0.84–1.08)
≥18 weeks	359 (7.5)	373 (8.2)	0.90 (0.77–1.05)
≥22 weeks	264 (5.5)	266 (5.8)	0.93 (0.78–1.12)
West Europe and the US			
<12 weeks	285 (3.1)	261 (2.9)	1.07 (0.92–1.25)
≥12 weeks	593 (6.4)	562 (6.3)	1.01 (0.90–1.14)
≥18 weeks	401 (4.4)	381 (4.3)	1.10 (0.95–1.28)
≥22 weeks	299 (3.3)	272 (3.1)	1.04 (0.87–1.25)
Canada			
<12 weeks	6,635 (1.0)	6,300 (1.0)	1.00 (0.96–1.03)
≥12 weeks	10,034 (1.6)	9,441 (1.6)	1.01 (0.98–1.04)
≥18 weeks	6,644 (1.0)	6,268 (1.0)	1.02 (0.98–1.06)
≥22 weeks	4,613 (0.7)	4,305 (0.7)	1.00 (0.95–1.04)

Findings were similar when we examined mother tongue (Table 5). Women with any foreign mother tongue who were pregnant with boys were 1.08 times more likely to marry after 18 weeks of gestation (95% CI 1.00–1.16), compared with women who were pregnant with girls. Women with Arabic or other neighbouring mother tongues who were pregnant with boys were 1.32 times more likely to marry after 18 weeks (95% CI 1.04–1.68), compared with women from the same region who were pregnant with girls.

**TABLE 5:** Association of child sex with gestational age at marriage according to mother tongue, Canada, 1990–2018.

	No. women who married during pregnancy (%)		Hazard ratio boys vs girls (95% confidence interval)
	Pregnant with boys N = 22,102	Pregnant with girls N = 20,797	
Any foreign language			
<12 weeks	1,275 (5.5)	1,239 (5.5)	0.99 (0.92–1.07)
≥12 weeks	2,489 (10.7)	2,301 (10.2)	1.06 (0.99–1.12)
≥18 weeks	1,701 (7.3)	1,543 (6.9)	1.08 (1.00–1.16)
≥22 weeks	1,216 (5.2)	1,072 (4.8)	1.07 (0.98–1.17)
Arabic and neighbouring Afro-asiatic languages^a^			
<12 weeks	214 (15.2)	182 (13.5)	1.15 (0.96–1.39)
≥12 weeks	252 (17.9)	211 (15.7)	1.17 (0.96–1.42)
≥18 weeks	175 (12.4)	136 (10.1)	1.32 (1.04–1.68)
≥22 weeks	130 (9.2)	93 (6.9)	1.32 (1.00–1.75)
Russian and Slavic languages^b^			
<12 weeks	115 (6.4)	95 (5.5)	1.12 (0.88–1.43)
≥12 weeks	295 (16.5)	279 (16.1)	1.05 (0.89–1.25)
≥18 weeks	218 (12.2)	197 (11.4)	1.05 (0.86–1.29)
≥22 weeks	147 (8.2)	139 (8.0)	1.05 (0.81–1.36)
Indo-Pakistani, Chinese, and related languages^c^			
<12 weeks	323 (7.6)	307 (7.3)	0.99 (0.85–1.14)
≥12 weeks	550 (12.9)	542 (13.0)	1.03 (0.91–1.17)
≥18 weeks	366 (8.6)	354 (8.5)	1.00 (0.85–1.17)
≥22 weeks	263 (6.2)	256 (6.1)	1.02 (0.84–1.22)
French or English			
<12 weeks	7,252 (1.1)	6,797 (1.1)	1.01 (0.98–1.04)
≥12 weeks	11,086 (1.7)	10,460 (1.7)	1.01 (0.98–1.04)
≥18 weeks	7,347 (1.1)	6,992 (1.1)	1.01 (0.98–1.05)
≥22 weeks	5,147 (0.8)	4,833 (0.8)	0.99 (0.95–1.04)

a Turkish, Egyptian, and other languages of Southwest Asia and North Africa.

b Czech, Estonian, Lettish, Lithuanian, Polish, Romanian, Slovak, Ukrainian, Yugoslav.

c Japanese, and other languages of Southeast Asia.

Restricting the analysis to primiparous women led to similar results although confidence intervals widened. There was no evidence of a change in associations over time.

## Discussion

In this study of 1.3 million women who were unmarried at conception, women originating from the Middle East, North Africa, and Eastern Europe who were pregnant with boys were more likely to marry during pregnancy than women who were pregnant with girls. The associations strengthened during the second trimester, when ultrasounds can more reliably detect the sex of the child. Child sex was not associated with the likelihood of marriage in women originating from Canada, West Europe and the United States, Asia, Sub-Saharan Africa, Latin America, and the Caribbean. These findings suggest that child sex among some cultural minorities may influence the likelihood of marriage during pregnancy. Marital status is a well-known determinant of maternal and infant health, and marriage during pregnancy may be an indicator of son preference and gender inequity that merits more attention in epidemiologic research [7, 8].

Gender inequity in maternal and child health is challenging to measure in Western settings. While many studies have examined sex and gender differences in physical and mental health [10, 20], gender inequity related to pregnancy and birth outcomes receives less attention. Most indicators of gender inequity rely on gross measures such as the World Health Organization gender inequality index (GII) and gender empowerment measure (GEM) which are based on education, income, professional positions, and political participation [21]. The GEM has a reproductive health component, but only includes maternal mortality ratios and adolescent birth rates [21]. In Quebec, gender inequity is measured by lower income and employment rates, and fewer women in leadership positions compared with men [22]. These indicators are difficult to adapt to reproductive health.

Much of the literature on gender inequity during pregnancy focuses on sex selection. Several studies have shown that countries such as India, China, South Korea, Azerbaijan, and Armenia have more than the expected 105 male births for every 100 female births [3–5]. These high sex ratios have been attributed to sex-selective abortion of female fetuses. There also is evidence that sex selection is practised among immigrant populations in Western countries [1, 2, 11]. In the 1990s, women of Indo-Pakistani descent in Quebec had a considerably elevated number of male births thought to be due to sex selection [11]. In settings where son preference has been documented, studies have shown that pregnant women who are expecting girls receive less prenatal care [23], and that girls are breastfed for a shorter period than boys [5].

In this study, women from selected cultural minorities, including the Middle East, North Africa, and Eastern Europe, had a greater likelihood of marriage if they were expecting boys. While very little literature is available on this form of gender inequity, two studies from the United States assessed marital inequality without examining differences between cultural groups [24, 25]. Women who were carrying boys were more likely to marry during pregnancy than women carrying girls, but only if they had an ultrasound during pregnancy [24]. Similarly, unmarried women who gave birth to boys were more likely to marry the child’s biological father after pregnancy than women who gave birth to girls [25]. Other research suggests that men in the United States are more likely to live with their children and less likely to divorce if they have sons [24], and that having a firstborn daughter increases the probability of living without a father [26].

Son preference is known to be influenced by cultural, religious, and societal factors [1, 2]. Some religions, including Islam, Christianity, and Jainism, limit the practice of abortion [1, 2, 6]. In communities with taboos against abortion, sex ratios at birth may not be good indicators of gender inequity or son preference because sex-selective abortions may be less accessible. Son preference may instead manifest as differences in prenatal and postnatal care, probability of having additional children, or family structure [1, 2]. This tendency may explain why we found a higher likelihood of marriage among women carrying boys from the Middle East, North Africa, and Eastern Europe—regions with large Islamic and Christian communities where abortions are less practiced [6, 27]. We found no such trend among women of South or East Asian origin, including China and India where son preference and sex-selective abortion have been documented [1, 5].

A growing body of literature suggests that marriage is beneficial to maternal and child health. Relative to unmarried women, married women have 32% lower risk of low birth weight, 18% lower risk of preterm birth, and 31% lower risk of small-for-gestational age birth [7]. The benefits appear to continue through childhood [9, 24, 28, 29]. Although children whose parents are in stable cohabitation also have better outcomes than children of single parents, the benefits are not as pronounced compared with married parents [9, 24]. Marriage increases the availability of resources, mental health, relationship quality, parenting quality, paternal involvement, and family stability [9, 30]. Marriage is associated with better cognitive performance and lower risk of behavioural problems, obesity, asthma, injury, and child hospitalization [9, 24, 28, 29]. A lower likelihood of marriage among women who are carrying girls may therefore have long-term implications for the health and development of daughters [9, 24, 25]. Attention to reducing sex-based disparities in marriage has potential to improve the health of children, especially daughters.

### Limitations

This study has limitations. We used an administrative registry where errors in the coding of exposures or outcomes cannot be ruled out. Mother tongue, country of origin, and date of marriage were self-reported and may be misclassified, possibly attenuating the results toward the null. Grouping diverse countries into broad regions of origin may have masked smaller cultural differences. We were unable to confirm that parents knew the sex of the child, or the exact gestational age at which sex was determined. Nondifferential misclassification of exposure is expected to bias associations towards the null. We did not know if the proportion of women who refused to know the sex of the child varied by region of origin. We lacked data on the reason for marriage, religion, residency status, duration of residence, and paternal region of origin. We could not account for sex-selective abortions that may have reduced the number of girls born during the study period. The conclusions may not generalize to settings where routine ultrasound is not used or to populations with considerably different characteristics or cultural norms.

### Conclusion

In this retrospective cohort study of women who were unmarried at the start of pregnancy, child sex was associated with the probability of marriage among several cultural minorities in a large Canadian province. Women from the Middle East, North Africa, and Eastern Europe who were carrying boys were more likely to marry during pregnancy than women who were carrying girls. The association was present throughout the pregnancy, particularly the second trimester when ultrasounds can inform parents of the sex of their child. The results suggest that child sex among some cultural minorities may influence the likelihood of marriage during pregnancy. Future studies should consider using marriage during pregnancy as an indicator of son preference. Careful thought should be given to developing other novel indicators and ways of measuring gender inequity in the immediate future.
